# The promiscuous enzyme medium-chain 3-keto-acyl-CoA thiolase triggers a vicious cycle in fatty-acid beta-oxidation

**DOI:** 10.1371/journal.pcbi.1005461

**Published:** 2017-04-03

**Authors:** Anne-Claire M. F. Martines, Karen van Eunen, Dirk-Jan Reijngoud, Barbara M. Bakker

**Affiliations:** 1 Laboratory of Pediatrics, University of Groningen, University Medical Center Groningen, The Netherlands; 2 Systems Biology Centre for Energy Metabolism and Ageing, University of Groningen, University Medical Center Groningen, The Netherlands; Chalmers University of Technology, SWEDEN

## Abstract

Mitochondrial fatty-acid beta-oxidation (mFAO) plays a central role in mammalian energy metabolism. Multiple severe diseases are associated with defects in this pathway. Its kinetic structure is characterized by a complex wiring of which the functional implications have hardly been explored. Repetitive cycles of reversible reactions, each cycle shortening the fatty acid by two carbon atoms, evoke competition between intermediates of different chain lengths for a common set of ‘promiscuous’ enzymes (enzymes with activity towards multiple substrates). In our validated kinetic model of the pathway, substrate overload causes a steep and detrimental flux decline. Here, we unravel the underlying mechanism and the role of enzyme promiscuity in it. Comparison of alternative model versions elucidated the role of promiscuity of individual enzymes. Promiscuity of the last enzyme of the pathway, medium-chain ketoacyl-CoA thiolase (MCKAT), was both necessary and sufficient to elicit the flux decline. Subsequently, Metabolic Control Analysis revealed that MCKAT had insufficient capacity to cope with high substrate influx. Next, we quantified the internal metabolic regulation, revealing a vicious cycle around MCKAT. Upon substrate overload, MCKAT’s ketoacyl-CoA substrates started to accumulate. The unfavourable equilibrium constant of the preceding enzyme, medium/short-chain hydroxyacyl-CoA dehydrogenase, worked as an amplifier, leading to accumulation of upstream CoA esters, including acyl-CoA esters. These acyl-CoA esters are at the same time products of MCKAT and inhibited its already low activity further. Finally, the accumulation of CoA esters led to a sequestration of free CoA. CoA being a cofactor for MCKAT, its sequestration limited the MCKAT activity even further, thus completing the vicious cycle. Since CoA is also a substrate for distant enzymes, it efficiently communicated the ‘traffic jam’ at MCKAT to the entire pathway. This novel mechanism provides a basis to explore the role of mFAO in disease and elucidate similar principles in other pathways of lipid metabolism.

## Author summary

Burning of fat is essential to provide energy to our bodies. However, fat metabolism is also involved in well-known diseases, such as metabolic syndrome and type 2 diabetes. In severe, inherited deficiencies of enzymes in the fatty-acid oxidation pathway, children run the risk of life-threatening low glucose levels or ‘hypoglycemia’. This typically occurs during overnight fasting, when the body depends on fat stores for its energy supply. To understand the sequence of events leading to hypoglycemia and eventually provide novel therapies, we simulated the process with a validated computer model. We found that when the supply of fat to the liver was increased beyond a critical value, the rate of fatty-acid oxidation declined. Under these circumstances, intermediate metabolites accumulated, very much like the situation in a traffic jam. The risk of a metabolic traffic jam was enhanced in fatty-acid oxidation disorders. In the body this would lead to insufficient energy to maintain blood glucose levels. In this paper we unravel the vicious cycle that leads to the detrimental flux decline and identify a critical role for the last enzyme of the pathway, MCKAT. Our results suggests that MCKAT or its regulators may be promising drug targets.

## Introduction

The mitochondrial fatty-acid oxidation (mFAO) in the liver is of key importance for the production of ATP and ketone bodies during fasting. It allows glucose to be spared for utilization in the periphery, can supply the energy for de novo glucose production, and provides precursors for ketone-body synthesis. Thus, mFAO is intricately connected with other pathways of energy metabolism. For almost all enzymes of the pathway, inborn deficiencies associated with human disease have been identified [1,2]. All these disorders share the risk of ‘hypoketotic hypoglycemia’, a life-threatening condition with low blood levels of glucose and ketone bodies [1,2]. The severity of the disease symptoms depends on which enzyme is missing, whether there is residual activity, but there is also an unexplained variability between individuals. For example, some children with a loss-of-function mutation in the enzyme medium-chain acyl-CoA dehydrogenase (MCAD) present with a life-threatening drop in blood glucose, while others with the same mutation never show any phenotype [3–6]. Moreover, MCAD-deficient children do oxidize medium-chain fatty acids normally under non-challenging conditions [7]. It seems therefore that not the oxidation capacity, but the capability to use this capacity under all circumstances, hence the robustness of the mFAO pathway, is compromised in these patients.

Recently, we constructed and experimentally validated a dynamic computational model of mFAO pathway in rat liver [8]. Model simulations showed that the pathway is intrinsically sensitive to becoming overloaded with its substrate palmitoyl-CoA, a sixteen-carbon (C16), saturated acyl-CoA derivative of the dietary fatty acid palmitic acid. At high substrate concentrations the mFAO flux declined, intermediate acyl-CoA esters accumulated and the acyl carrier coenzyme A (CoA) was depleted. Subsequent simulations of mFAO in MCAD-deficient mouse liver [9], based on detailed experimental characterization of these mice, showed that the enzyme deficiency reduced the tolerated substrate concentration and aggravated the risk of overload [9]. We hypothesize that in patients with a mFAO disorder a similar phenomenon may be induced during fasting, due to the recruitment of fatty acids from adipose stores, possibly in combination with accidental perturbations of other CoA-dependent processes. Related to this, it has been proposed that an imbalance between fatty acid uptake into the mitochondria and mFAO, accumulation of mFAO intermediates, and incomplete mFAO lie at the basis of insulin resistance in the muscle [10]. To get more insight into the mechanisms underlying these different diseases, it is important to understand how the imbalance arises, and the computational model gives us some first hints, as outlined below.

**Fig 1:**
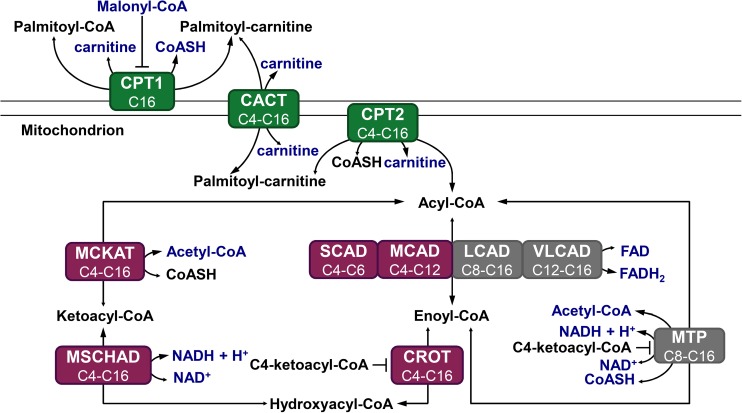
Schematic representation of the modelled mFAO pathway in rat. Metabolites in blue are fixed parameters, while other metabolites are free variables. The sum of variable CoA esters and free CoA is a conserved moiety. Green: enzymes participating in the carnitine cycle; purple: enzymes participating in the short-chain branch; grey: enzymes participating in the medium-and long-chain branch. All processes are reversible and the size of the arrowheads indicates the net direction of the flux. This model is exactly the same as published before [8], without modifications.

A special feature of the mFAO biochemistry is the promiscuity of its enzymes, meaning that each enzyme catalyses the conversion of fatty-acid derivatives of a range of chain lengths [2,8]. Oxidation of a long-chain fatty acid requires uptake into the mitochondrial matrix by the acyl-carnitine shuttle and multiple rounds of mFAO (Fig 1). In each round, the acyl-CoA ester is shortened by two carbon atoms and one acetyl-CoA molecule is produced. Each cycle consists of four consecutive reactions catalysed by acyl-CoA dehydrogenase (E.C. 1.3.99.3 and E.C.1.3.8.1), enoyl-CoA hydratase or crotonase (CROT, E.C. 4.2.1.74 and E.C. 4.2.1.150), hydroxyacyl-CoA dehydrogenase (E.C. 1.1.1.211 and E.C. 1.1.1.35) and ketoacyl-CoA thiolase (E.C. 2.3.1.16)). Four isoenzymes (the Very-Long-, Long-, Medium-, and Short-Chain Acyl-CoA Dehydrogenases VLCAD, LCAD, MCAD, and SCAD) catalyse the first reaction of the cycle, accepting a range of acyl-CoA substrates of different, overlapping chain lengths. Mitochondrial Trifunctional Protein (MTP) then catalyses the next three reactions for substrates of 8 carbon atoms or longer, while shorter substrates (C6 and C4) are exclusively converted by a distinct CROT, medium/short-chain hydroxyacyl-CoA dehydrogenase (M/SCHAD), and medium-chain ketoacyl-CoA thiolase (MCKAT). Since each enzyme is promiscuous and catalyses reactions in multiple rounds, the different substrates and resulting products compete for binding to the active site.

In the construction of the model [8], the total concentration of CoA has been considered as a conserved moiety, i.e. free CoA and CoA esters are only interconverted and CoA synthesis is outside the scope of the model. All reactions were treated as reversible and product sensitive. Taking into account the promiscuity of the enzymes, reversible Michaelis-Menten equations were modified to include all CoA esters that an enzyme can produce or consume as competitive inhibitors, with their affinity constant *K*_*m*_ serving as competitive inhibition constant. An example of such a rate equation, for the conversion of C16-acyl-CoA by the enzyme VLCAD, is:

$$v_{VLCAD,C16}=\frac{sf_{VLCAD,C16}\cdot V_{max,VLCAD}\cdot \left(\frac{\left[C16-acyl-CoA\right]}{Km_{VLCAD,C16-acyl-CoA}}\cdot \frac{\left[FAD\right]}{Km_{VLCAD,FAD}}-\frac{\left[C16-enoyl-CoA\right]}{Km_{VLCAD,C16-acyl-CoA}}\cdot \frac{\left[FADH_{2}\right]}{Km_{VLCAD,FAD\cdot {Keq}_{VLCAD}}}\right)}{\left(1+\frac{\left[C16-acyl-CoA\right]}{Km_{VLCAD,C16-acyl-CoA}}+\underset{inhibition\;by\;reaction\;product}{\underset{︸}{\frac{\left[C16-enoyl-CoA\right]}{Km_{VLCAD,C16-enoyl-CoA}}}}+\underset{inhibition\;by\;substrates\;and\;products\;of\;other\;chain\;lengths}{\underset{︸}{\sum_{n=12}^{n=14}\frac{\left[Cn-acyl-CoA\right]}{Km_{VLCAD,Cn-acyl-CoA}}+\frac{\left[Cn-enoyl-CoA\right]}{Km_{VLCAD,Cn-enoyl-CoA}}}}\right)\cdot \left(1+\frac{\left[FAD\right]}{Km_{VLCAD,FAD}}+\frac{\left[FADH_{2}\right]}{Km_{VLCAD,FADH_{2}}}\right)}$$ (1)

In this equation, the metabolites in the grey box are the substrates and products of competing reactions and act as inhibitors. If this promiscuity was removed *in silico* by taking out all competitive inhibition terms except direct product inhibition (i.e. all terms in the grey box), the flux exhibited normal saturation behaviour. This suggests that the enzyme promiscuity and the resulting metabolic competition cause the flux decline at high substrate concentrations [8,9]. *How* competition mediates this emergent and potentially deleterious flux decline phenomenon has, however, not been studied yet. To be able to explore its role in disease and get a better insight into the normal functioning of the mFAO pathway, it is important to unravel the complete sequence of events. This will be crucial to understand how a healthy mFAO is protected against this phenomenon on the one hand and how the risk for patients can be minimized on the other hand.

The aim of this study is to dissect the mechanism via which enzyme promiscuity and the resulting metabolic competition induces flux decline at high substrate concentrations. Specifically, we will dissect (i) the role of promiscuity of each individual enzyme and (ii) the role of each of the metabolites in the internal regulation of the pathway. To this end we use computational modelling and metabolic control analysis [11], and quantify the regulatory strength of the metabolites by a previously published method [12]. We will reveal a vicious cycle that starts at the very end of the pathway around MCKAT and is amplified and transmitted throughout the entire pathway. We will discuss how natural regulation and proper embedding in surrounding pathways may prevent this phenomenon in healthy individuals and how it may potentially be attenuated or aggravated in disease.

## Results

### Description of the phenotype

**Fig 2:**
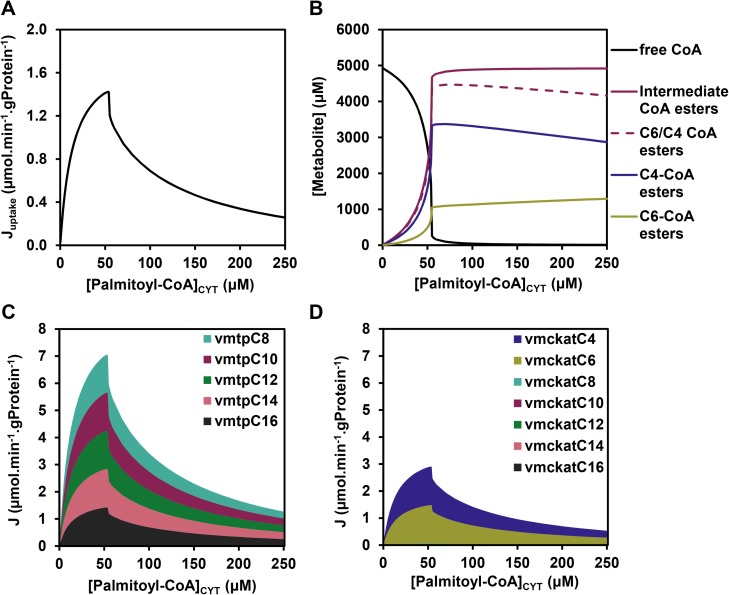
Simulated steady-state fluxes and concentrations in the mFAO model. **(A)** The effect of cytosolic palmitoyl-CoA concentration ([palmitoyl-CoA]_CYT_) on the steady-state flux. The steady-state uptake flux of palmitoyl-CoA (J_uptake_) is plotted (calculated as the steady-state flux of palmitoyl-carnitine through CACT, i.e. the uptake of palmitoyl-carnitine into the mitochondria), in contrast to van Eunen *et al*. 2013 [8] in which the NADH flux was plotted. The two are uniquely linked with the latter being 7-fold higher. **(B)** The steady-state concentrations of free CoA (identical to data from [8]), the sum of all intermediate CoA esters, the sum of all C4- and C6 CoA esters and the subset of C4- and C6 CoA esters (new results). **(C-D)** Distribution of steady-state fluxes (J) of different chain-length substrates through MTP and MCKAT, respectively. Only the chain lengths which are converted by these enzymes (cf. Fig 1) are included.

In our previous studies we showed that the flux decline at high cytosolic palmitoyl-CoA concentrations ([palmitoyl-CoA]_CYT_) (Fig 2A) was due to the depletion of the free CoA pool, which resulted from the accumulation of CoA esters (Fig 2B). We first calculated which CoA esters accumulated, and these turned out to be predominantly the CoA esters of chain lengths C4 and C6 (Fig 2B). From enoyl-CoA onward the mFAO pathway consists of two branches: the MTP branch (grey in Fig 1), which exclusively converts the longer chain lengths of C8 and higher, and the broad specificity CROT-M/SCHAD-MCKAT branch (purple in Fig 1), which covers the complete range of chain lengths from C4 through C16. Thus, the C4- and C6 CoA esters can only be oxidized via the CROT-M/SCHAD-MCKAT branch, while longer chain lengths can be oxidized via both branches. However, the actual flux of C8 and longer enoyl-CoA esters went almost exclusively via the MTP route (Fig 2C). Only C4 and C6 CoA esters, which had no other option, took the CROT-M/SCHAD-MCKAT branch (Fig 2D). Therefore, we refer to the latter as the short-chain branch. The fact that precisely the esters in the short-chain branch accumulated was a first indication for a bottleneck in this branch.

### The flux decline originates at the promiscuous MCKAT enzyme

**Fig 3:**
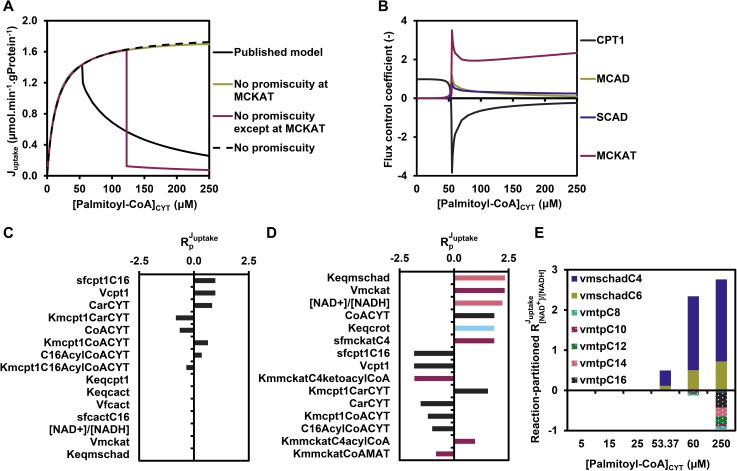
The role of the short-chain branch. **(A)** Steady-state flux versus [palmitoyl-CoA]_CYT_ in the published model (black line), a model without any enzymatic promiscuity (black dashed line, reproducing results from [8] as a reference), a model without promiscuity of MCKAT (green line), and finally a model with only promiscuity of MCKAT, but not of any of the other enzymes (purple line). **(B)** The flux control coefficients in the original model as a function of [palmitoyl-CoA]_CYT_. Only enzymes with a substantial contribution are included; for a complete set of flux control coefficients, see figure A in S2 Fig. **(C-D)** Top 15 flux response coefficients with the highest absolute values at 25 μM (C) and 60 μM (D) of [palmitoyl-CoA]_CYT_. The parameters *p* for which the flux response coefficient was calculated is indicated on the Y-axis. Dark grey bars represent CPT1-related parameters, pink bars represent M/SCHAD-related parameters, purple bars represent MCKAT-related parameters and the light blue bar represents a crotonase-related parameter. **(E)** Flux response coefficient of [NAD^+^]/[NADH] ($R_{\left[{NAD}^{+}]/[NADH\right]}^{Juptake}$) partitioned in contributions of individual NAD^+^-dependent reactions at 5 palmitoyl-CoA concentrations according to $R_{\left[{NAD}^{+}]/[NADH\right]}^{J}=\sum_{i}C_{i}^{J}∙\varepsilon_{\left[{NAD}^{+}]/[NADH\right]}^{v_{i}}$. (cf. Eq 4). The contributions of M/SCHAD reactions C8-C16 to $R_{\left[{NAD}^{+}]/[NADH\right]}^{Juptake}$ were negligible and therefore not shown in the legend.

To gain more insight into the mechanism via which enzyme promiscuity induces the flux decline, we first dissected the role of promiscuity of each individual enzyme. To this end, enzyme promiscuity was removed computationally–either from all enzymes simultaneously or from each enzyme individually–by partitioning an enzyme pool to distinct fractions dedicated to a single chain length (see Methods). In the non-promiscuous rate equation, the enzyme was still inhibited by its own product, but not by products and substrates of other chain lengths. At the same time the total catalytic capacity (*V*_*max*_) was partitioned over the different reactions catalysed by each enzyme (see Methods for a mathematical description). The flux decline in the published model with full promiscuity was plotted as a reference (Fig 3A). If promiscuity was removed from all enzymes simultaneously, the flux did not decline anymore, but approached a maximum at saturating palmitoyl-CoA concentration (Fig 3A). In contrast, removing the promiscuity from any enzyme individually did not prevent flux decline (figure A in S1 Fig), except when promiscuity was removed from the MCKAT reaction (Fig 3A). Conversely, when promiscuity was removed from all enzymes *except* MCKAT, the flux dropped even more steeply than in the original model with full promiscuity (Fig 3A). These results show that–under the given conditions—promiscuity of MCKAT is both necessary and sufficient for the flux decline at a critical concentration of about 50 μM of palmitoyl-CoA. Of note, when the palmitoyl-CoA concentration was increased to supraphysiological concentrations above 250 μM, even the model lacking MCKAT promiscuity exhibited a flux decline (figure B in S1 Fig). In the model with promiscuity in MCKAT only, the flux dropped at a higher palmitoyl-CoA concentration than in the model with full promiscuity (Fig 3A). Together, this demonstrates that the promiscuity of the other enzymes could in principle contribute to flux decline, but under physiological conditions the promiscuity of MCKAT is by far the most important.

### MCKAT becomes the most important flux-controlling enzyme when the flux declines

Palmitoyl-CoA is the substrate of CPT1, the first enzyme of the transport shuttle, consisting of CPT1, CACT, and CPT2 (Fig 1). The decline of flux as a function of palmitoyl-CoA suggests that CPT1 activity has a negative control over the pathway flux. To investigate how the flux is controlled under these conditions, the control exerted by each enzyme over the flux was quantified. In Metabolic Control Analysis [11,13–15], the flux control coefficient $C_{i}^{J}$ of an enzyme *i* over the steady-state flux *J* is defined as:

$$C_{i}^{J}=\frac{dlnJ/dp}{\partial lnv_{i}/\partial p}$$ (2)

in which *p* is a parameter that only affects the rate *v* of enzyme *i*. A flux control coefficient equal to 1 means that the direct effect of *p* (e.g. the enzyme concentration or *V*_*max*_) on the enzyme is fully translated to the steady-state flux. If the absolute control coefficients of the other enzymes are close to zero, this indicates that the enzyme is completely rate-controlling. A low flux control coefficient indicates that the primary effect of *p* is counteracted by adaptations of metabolite concentrations, such that the final effect on the steady-state flux is small. The results are shown in Fig 3B. At low palmitoyl-CoA concentrations CPT1 fully controlled the flux, as was apparent from its flux control coefficient equal to 1, while all other flux control coefficients were negligible (Figs 3B and S2A). At higher palmitoyl-CoA concentrations, however, the flux control coefficient of CPT1 became strongly negative, indeed indicating that a stimulation of its catalytic capacity reduced the flux steeply. According to the summation theorem [11,14] the sum of all flux control coefficients is equal to 1, implying that the negative control exerted by CPT1 should be compensated by high positive flux control coefficients of one or more other enzymes. The major share of this positive flux control was adopted by MCKAT, and a minor part by MCAD and SCAD (Figs 3B and S2A). Dissecting the flux control coefficient of MCKAT in that of individual, chain-length-specific reactions showed that only its C4 and C6 reactions contributed substantially (figure B in S2 Fig), again demonstrating a pivotal role of the short-chain branch of the pathway. Other flux control coefficients were small under all conditions (figure in S2 Fig).

### The accumulation of intermediates is amplified by the short-chain branch

We then wondered whether other reactions, besides MCKAT, CPT1, MCAD, and SCAD modulated the phenotype. Therefore, we quantified the flux response coefficients for all parameters *p* in the model at two cytosolic palmitoyl-CoA concentrations: below the critical concentration for flux decline (25 μM) and beyond this critical concentration when the flux is below its maximum capacity (60 μM). The flux response coefficient $R_{p}^{J}$ [15] quantifies the relative effect of a small change of a parameter *p* on the steady-state flux according to:

$$R_{p}^{J}=dlnJ/dlnp$$ (3)

Fig 3C and 3D show the top 15 parameters with the highest absolute flux response coefficients at 25 μM and 60 μM of palmitoyl-CoA, respectively. In agreement with the high flux control of CPT1, the eight highest response coefficients at 25 μM comprised exclusively parameters that affect the rate of CPT1, including its (fixed) substrate- and product concentrations (Car_CYT_, CoA_CYT_, C16AcylCoA_CYT_), catalytic capacity (sf_CPT1,C16_ and V_cpt1_), and affinity constants (K_m,CPT1,CarCYT_, K_m,CPT1,CoACYT_, K_m,CPT1,C16AcylCoACYT_) (Fig 3C), and the next response coefficients were already too low to visualize. At 60 μM, however, anticipated parameters relating to MCKAT appeared (i.e. V_MCKAT_, sf_MCKAT,C4_, K_m,mckat,C4-ketoacyl-CoA_, K_m,mckat,C4-acyl-CoA_ and K_m,CoA,MAT_, as shown by the purple bars). In addition, the [NAD^+^]/[NADH] ratio in the mitochondrial matrix and the equilibrium constant of M/SCHAD (shown by the pink bars) and CROT (shown by the light blue bar) were in the top 4 of highest response coefficients (Fig 3D).

Since NAD^+^ is a substrate of M/SCHAD, it seems likely that the high flux response coefficients of [NAD^+^]/[NADH] and the equilibrium constant are mutually related. We therefore set out to elucidate the mechanism via which they affected the flux. The role of NAD^+^ is complex, since it is also a substrate for the MTP reaction and both M/SCHAD and MTP catalyse reactions of substrates with a range of fatty-acid chain lengths. Therefore, we dissected the response coefficient of [NAD^+^]/[NADH] into contributions of all the different reactions that are influenced by this ratio, based on the combined response theorem [15]:

$$R_{\left[{NAD}^{+}]/[NADH\right]}^{J}=\sum_{i}C_{i}^{J}∙\varepsilon_{\left[{NAD}^{+}]/[NADH\right]}^{v_{i}}$$ (4)

in which the summation is over all reactions *i*, and $\varepsilon_{NAD+/NADH}^{i}$ denotes the elasticity (i.e. the direct sensitivity) of the rate of reaction *i* towards the NAD^+^-NADH ratio, according to:

$$\varepsilon_{\left[{NAD}^{+}\right]/\left[NADH\right]}^{v_{i}}=\partial lnv_{i}/\partial ln\left([{NAD}^{+}\right]/\left[NADH]\right)$$ (5)

The dissection of Eq 4 in its reaction-specific terms $C_{i}^{J}∙\varepsilon_{\left[{NAD}^{+}]/[NADH\right]}^{v_{i}}$ (Fig 3E) revealed two large and positive terms, representing the C4 and C6 reactions catalysed by M/SCHAD. The terms representing MTP were small and negative. We conclude from this that the positive effect of [NAD^+^]/[NADH] on the flux can be attributed exclusively to the short-chain reactions (C4 and C6) of M/SCHAD, while MTP even exerted a small negative contribution. The M/SCHAD reaction is close to thermodynamic equilibrium when the pathway is at steady state (cf. the similar shapes of M/SCHAD substrates and products in figure C and D in S3 Fig, as opposed to those of MCKAT substrates and products in figure D and A in S3 Fig). An increase of the [NAD^+^]/[NADH] ratio shifts the equilibrium of the reaction in the forward direction, explaining why it has the same effect as a change of the equilibrium constant. The reason that the equilibrium constants are so important for the flux is that the equilibrium constant of M/SCHAD is extremely unfavourable at 2.17·10^−4^ (dimensionless, cf. Fig 1) in the forward direction. A small accumulation of C4- and C6 ketoacyl-CoA, the direct substrates of MCKAT, will therefore entrain the hydroxyacyl-CoA substrates of M/SCHAD to accumulate several orders of magnitude more, the exact ratio depending on the [NAD^+^]/[NADH] ratio (Figure C in S3 Fig). The equilibrium constant of CROT is 3.13, thus inducing an enoyl-CoA accumulation of almost the same order of magnitude (Figure B in S3 Fig). Thus, M/SCHAD and CROT amplify the accumulation of CoA esters enormously, thereby draining the free CoA pool.

Finally, in Fig 3D only MCKAT parameters related to the C4 reaction appear (i.e. sf_MCKAT,C4_, K_m,mckat,C4-ketoacyl-CoA_), but hardly any parameters related to the C6 reaction, except for the affinity toward its product C4-acyl-CoA (i.e. K_m,mckat,C4-acyl-CoA_), since the latter have lower response coefficients. This may be attributed to differences in the catalytic constants between the C4 and C6 reactions, notably (i) the C4-specific MCKAT catalytic capacity (sf_MCKATC4_, a chain-length-specific modifying factor for V_max_) being 2-fold lower than its C6-specific counterpart (sf_MCKATC6_) (0.49 versus 1) and (ii) the almost 2-fold lower affinity of MCKAT for its C4-ketoacyl-CoA substrate than for its C6-ketoacyl-CoA substrate, quantified by a 2-fold higher corresponding Michaelis-Menten constant (K_m_) (12.4 versus 6.7 μM).

### Internal regulation reveals a vicious cycle around MCKAT

**Fig 4:**
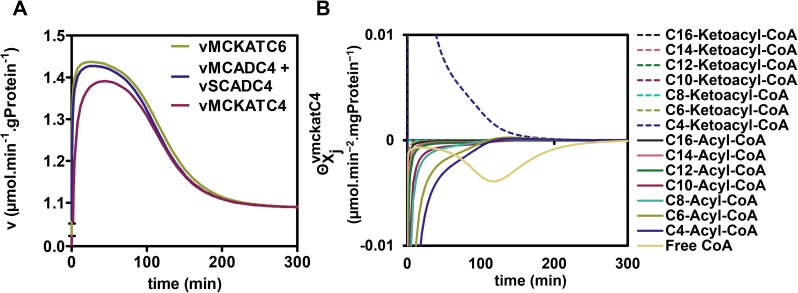
Time course of key short-chain reaction rates and regulation of the MCKAT-C4 reaction. **(A)** Time course of the reaction rates of MCKAT for its C6 and C4 substrates (vMCKATC6 and vMCKATC4, respectively) and of the summed activities of SCAD and MCAD on their C4 substrate (vMCADC4 + vSCADC4) after a sudden upshift of [palmitoyl-CoA]_CYT_ from 0.1 to 60 μM. **(B)** Time course of regulatory metabolite contributions to vMCKATC4 after a sudden [palmitoyl-CoA]_CYT_ increase from 0.1 to 60 μM.

To understand the sequence of events leading to flux decline and the potential role of competitive inhibition therein, we inspected time courses of metabolite concentrations and enzyme rates. Starting from a steady state at 0.1 μM, the concentration of the substrate palmitoyl-CoA was increased at time point zero to 25 or 60 μM and the response of the pathway was followed until the system relaxed into a new steady state. At both substrate concentrations metabolites accumulated or declined gradually, with hardly any detectable over- or undershoots (Figure A in S4 Fig). At a low concentration of palmitoyl-CoA (25 μM) the rates of all enzymes also increased gradually in time (Figure in S5 Fig). At 60 μM, however, the pattern was conspicuously different, as exemplified by enzyme rates around the C4 metabolites (Fig 4A). The rate of MCKAT on its C4-ketoacyl-CoA substrate lagged substantially behind the rate at which the C4-acyl-CoA entered the short-chain branch via SCAD and MCAD. Both rates increased in time as their substrate pools filled, until at time point 28 min, when they started to decline. The same happened at the level of the C6 reactions of MCAD/SCAD and MCKAT, albeit to a lesser extent (Figure in S6 Fig). This suggested that one or more of the metabolites inhibited these enzymes or became limiting.

We wondered which metabolites caused the enzyme rates to decline. Since each reaction rate depends on the concentrations of multiple primary substrates and products as well as competing metabolites of other chain lengths, the contribution of each individual metabolite to the regulation of the reaction rates was quantified, according to the method of Bruggeman et al. 2005 [12]. The concept behind this analysis is that a change in enzyme rate *v* is the mathematical product of a change in a metabolite concentration *X*_*j*_ multiplied by the elasticity of the enzyme rate to this metabolite concentration, and summed over all metabolites *j* that affect the enzyme:

$$\frac{dlnv}{dt}(t)=\sum_{j}\frac{\partial lnv}{\partial lnX_{j}}(t)∙\frac{dlnX_{j}}{dt}(t)\equiv \sum_{j}\Theta_{X_{j}}^{v}(t)$$ (6A)

with:

$$\Theta_{X_{j}}^{v}(t)\equiv \frac{\partial lnv}{\partial lnX_{j}}(t)∙\frac{dlnX_{j}}{dt}(t)$$ (6B)

Integration over the entire relaxation time from one steady state to another allows the dissection of the overall regulation of the rate into average contributions ${\bar{\Theta }}_{j}$ of each individual metabolite *X*_*j*_:

$$\frac{1}{t}\int_{0}^{t}\frac{dlnv}{dt}(\tau )d\tau =\sum_{j}\frac{1}{t}\int_{0}^{t}\frac{\partial lnv}{\partial lnX_{j}}(\tau )∙\frac{dlnX_{j}}{dt}(\tau )d\tau =\sum_{j}\frac{1}{t}\int_{0}^{t}\Theta_{X_{j}}^{v}(\tau )d\tau =\sum_{j}{\bar{\Theta }}_{X_{j}}^{v}$$ (7)

The average absolute contribution of each metabolite is given by:

$${\left|\bar{\Theta }\right|}_{X_{j}}^{v}=\frac{1}{t}\int_{0}^{t}|\Theta_{X_{j}}^{v}(\tau )|d\tau$$ (8)

Fig 4B shows the contribution $\Theta_{X_{j}}^{v}(t)$ of all metabolites that affect the conversion of C4-ketoacyl-CoA by MCKAT plotted as a function of time for a transition from 0.1 to 60 μM of palmitoyl-CoA, calculated according to Eq 6B. The absolute area under each curve divided by the relaxation time is ${\left|\bar{\Theta }\right|}_{X_{j}}^{vMCKAT-C4}$ and denotes the average absolute contribution of a specific metabolite *j* to the change of the rate of the C4 reaction of MCKAT during the relaxation to the new steady state. A large positive contribution is made by the substrate C4-ketoacyl-CoA. Negative contributions were made by acyl-CoA esters of various chain lengths, which are the products of all the other reactions catalysed by MCKAT. Notably also the free CoA concentration made a substantial negative contribution. Free CoA is a substrate of MCKAT and therefore its elasticity coefficient $\frac{{\partial lnv}_{MCKAT-C4}}{\partial ln[CoA]}$ is positive, but since the concentration decreased with time, the overall contribution became negative.

**Fig 5:**
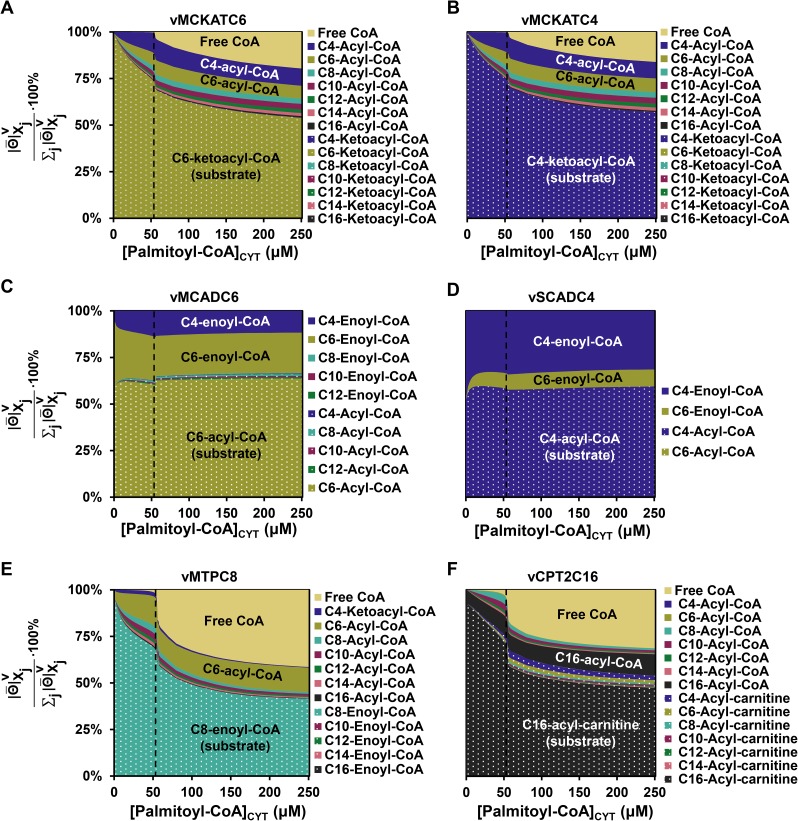
Regulation of key reactions by their substrates and products. **(A-F)** Relative average absolute contribution, i.e. $\frac{{\left|\bar{\Theta }\right|}_{X_{j}}^{v}}{\sum_{j}{\left|\bar{\Theta }\right|}_{X_{j}}^{v}}∙100\%$, of metabolites to the transition from the steady state at 0.1 μM to the indicated concentration of palmitoyl-CoA, calculated for vMCKATC6 (panel A), vMCKATC4 (panel B), vMCADC6 (panel C), vSCADC4 (panel D), vMPTC8 (panel E), vCPT2C16 (panel F).

In Fig 5A–5F these average contributions were plotted as a function of the substrate palmitoyl-CoA for a transition from 0.1 μM to the indicated concentration of palmitoyl-CoA. We started from the MCKAT reactions for its substrates C6 and C4-ketoacyl-CoA, at which the flux decline appeared to originate (Fig 5A and 5B). Like for the transition from 0.1 to 60 μM, also at other palmitoyl-CoA concentrations the MCKAT reaction was mostly regulated by the ketoacyl-CoA substrate of the reaction that is inspected (i.e. the C4-ketoacyl-CoA for the C4-MCKAT reaction and the C6-ketoacyl-CoA for the C6-MCKAT reaction), its co-substrate free CoA, and acyl-CoA esters of various chain lengths, including the acyl-CoA product of the reaction itself. The only overall positive contribution was by the ketoacyl-CoA substrate, due to the increase of its concentration in time (cf S7 Fig). The decrease of the co-substrate CoA yielded a negative contribution. The role of the acyl-CoA esters is paradoxical. They are products of MCKAT and therefore inhibit the enzyme, as can be seen from the negative regulatory contribution of the acyl-CoA esters in Fig 4B. At the same time the C6- and C4-acyl-CoA esters are substrates for the short-chain branch and are converted to C6- and C4-ketoacyl substrates for MCKAT.

**Fig 6:**
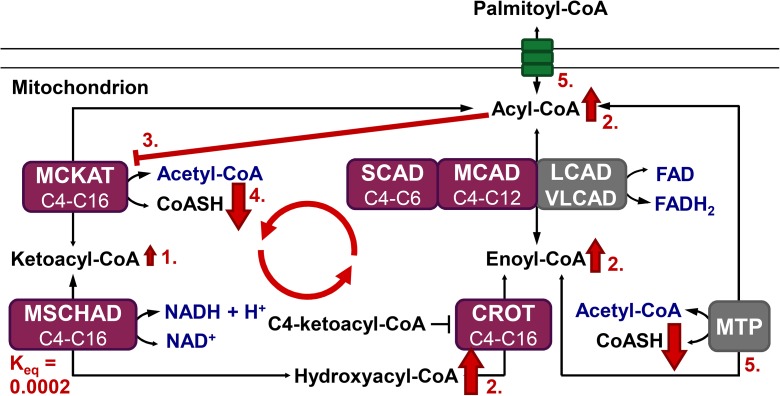
Elucidating the mechanism of flux decline. **1.** Upon substrate overload with cytosolic palmitoyl-CoA, MCKAT’s ketoacyl-CoA substrates start to accumulate. **2.** The unfavourable equilibrium constant of the preceding enzyme, medium/short-chain hydroxyacyl-CoA dehydrogenase, works as an amplifier, leading to the accumulation of upstream CoA esters, including acyl-CoA esters. **3.** These acyl-CoA esters are at the same time products of MCKAT and inhibit its already low activity further. **4.** Finally, the accumulation of CoA esters leads to a sequestration of free CoA. CoA being a cofactor for MCKAT, its sequestration limited the MCKAT activity even further, thus completing the vicious cycle. **5**. Since CoA is also a substrate for distant enzymes (such as MTP), it efficiently communicates the ‘traffic jam’ at MCKAT to the entire pathway.

By combining all data, we deduced the following mechanism (summarized in Fig 6). An upshift of palmitoyl-CoA fills the acyl-CoA pools, which are converted into enoyl-CoA esters and largely follow the MTP route down to C6-acyl-CoA. C6- and C4-acyl-CoA then follow the short-chain branch providing substrate to MCKAT, which has insufficient capacity to metabolise them at the same rate. This leads to an accumulation of MCKAT’s substrates C4- and C6-ketoacyl-CoA. As we have seen, the absolute concentrations of the ketoacyl-CoA esters are small, but their effects are amplified by equilibrating with hydroxyacyl-CoA esters and enoyl-CoA esters. These inhibit the acyl-CoA dehydrogenases and thus the upstream acyl-CoA esters start to accumulate. These acyl-CoA esters are at the same time products of MCKAT and therefore inhibit this enzyme. This aggravates the bottleneck at MCKAT. Moreover, the accumulating CoA esters sequester the free CoA and the overall result is a drastic reduction of the free CoA pool. Since CoA is a co-substrate of MCKAT, this limits the MCKAT reaction even further and traps the pathway in a vicious cycle.

In the time course (Fig 4A) the decrease of the rate sets in when the negative contributions of CoA decline and acyl-CoA accumulation surpass the positive contribution of the increased ketoacyl-CoA substrate (Fig 4B). To support the notion of MCKAT inhibition by the acyl-CoA esters, the occupancy of MCKAT with products and substrates of different chain-lengths was calculated (Figure A in S8 Fig). Indeed, the dominant occupants of the active site were C4- and C6-acyl-CoA, together occupying 66.4% of the available active sites at 60 μM of palmitoyl-CoA (Figure A in S8 Fig). Albeit individually less important, collectively the other acyl-CoA’s occupied 30.4% of the active sites under the same condition. At that point, the ketoacyl-CoA substrates occupied only 1.6% altogether.

Finally, we wondered how the clogging of metabolites in the short-chain branch was transmitted through the network to eventually slow down the entire pathway. To this end the internal metabolic regulation of MCAD, SCAD, MTP and CPT2 were computed. MCAD and SCAD, the enzymes at the gateway of the short-chain branch, responded by a strong regulation by their products C4-and C6-enoyl-CoA (Fig 5C and 5D). These regulations are overall negative around the point of flux decline, since their concentrations increase (Figure B in S3 Fig) and they inhibit the enzyme. This regulation was supported by the fact that the active sites of MCAD and SCAD were predominantly occupied by their products C4- and C6-enoyl-CoA (Figure B and C in S8 Fig). The acyl-CoA substrates of MCAD and SCAD occupied only a minor fraction of the active sites at steady state. More distant enzymes MTP and CPT2 responded to their local substrates and products, but also strongly via the connecting metabolite free CoA (Fig 5E and 5F).

## Discussion

The non-linear dynamic model used in this paper is the most structured and quantitative representation of our collective knowledge on mFAO. A computational model is completely transparent, a property that we exploited to dissect a vicious cycle leading to flux decline upon substrate overload (Fig 6). Using Metabolic Control Analysis and related approaches, we identified a pivotal role for the last enzyme of the pathway, MCKAT, for sequestration of CoA and competitive inhibition of MCKAT by its acyl-CoA products. This finding is in sharp contrast to our earlier speculation that the mutual competition between enzyme *substrates* might cause the flux decline [8].

The elucidated mechanism may have medical implications, both for patients with inborn defects in the mFAO and for multifactorial diseases in which mFAO is implicated. We have previously shown that MCAD deficiency makes the mFAO pathway susceptible to become overloaded by a high substrate concentration [9]. This can be understood in the light of the vicious cycle. In itself, the MCAD enzyme is not essential, since reactions catalysed by MCAD are taken over by SCAD, LCAD, and VLCAD (rodents, Fig 1) or by SCAD and VLCAD (humans) [16]. However, the latter acyl-CoA dehydrogenases have a weaker affinity for the medium-chain acyl-CoA esters than MCAD [17,18], thereby enhancing acyl-CoA accumulation and product inhibition of MCKAT. Analogously, the vicious cycle may play a role in obesity-related metabolic diseases, where a mitochondrial substrate overload has been proposed to lead to a metabolic gridlock [19]. The vicious cycle would definitely enhance the risk of such metabolic complications of high circulating fatty-acid concentrations in obesity.

In this section we will use our new insights to better understand how the pathway structure may play a role in disease, particularly regarding the lack of robustness of glucose and ketone-body homeostasis in patients with inherited mFAO defects. Products of the mFAO are required as energy source for *de novo* glucose production and as substrate for ketogenesis. We hypothesize therefore that the steep flux decline upon substrate overload plays an important role in this metabolic fragility. Key elements of the elucidated vicious cycle are (i) the high flux control of MCKAT and product inhibition of this enzyme by a competing set of acyl-CoA esters, leading to ketoacyl-CoA accumulation; (ii) the amplification of CoA-ester accumulation by M/SCHAD and the role of the [NAD^+^]/[NADH] ratio therein; and (iii) the resulting depletion of the free CoA concentration which further limits the MCKAT activity. We will structure our discussion around these three elements and their connection to the surrounding metabolic network. This will give insight into how perturbations in the surrounding metabolic network may stabilize or destabilize the pathway and thereby attenuate or aggravate the risk of metabolic failure.

*MCKAT–*The elucidated vicious cycle started at MCKAT. At low palmitoyl-CoA substrate concentrations the first enzyme of the pathway, CPT1 exerted a high flux control, in agreement with measurements in rat liver [20,21]. However, once the pathway became overloaded with palmitoyl CoA, MCKAT took over control over the steady-state flux in the model. In a comprehensive literature review about control of the β-oxidation pathway, Eaton [21] showed that MCKAT might indeed limit the mFAO flux in liver mitochondria under certain conditions. This conclusion was based on studies with MCKAT inhibitors which were found to affect the mFAO flux [22,23]. An experimental quantification of flux control showed that MCKAT started to control the flux only at high substrate concentrations [24]. This confirms our result that control shifts depending on the substrate concentration and thereby gives further credibility to the computational model.

Recently, the mitochondrial Shc isoform p46Shc was identified as a specific, endogenous inhibitor of MCKAT [25]. Shc knockdown increased mitochondrial palmitate oxidation, further corroborating the notion that MCKAT may limit the flux *in vivo*. Interestingly, Shc knockdown also led to a tendency of increased lifespan in mice [25] and the corresponding *SHC1* locus has been associated with longevity in man. Based on our results, this may be due to increased mFAO per se or to a relief of (toxic) CoA-ester accumulation. It will be interesting to investigate if the expression of the genes encoding MCKAT (ACAA2) or its inhibitor *SHC1* might differentiate between MCAD-deficient patients that presented with hypoketotic hypoglycemia and those that never did.

*M/SCHAD and the mitochondrial redox state–*The mitochondrial [NAD^+^]/[NADH] ratio connects the mFAO pathway to the respiratory chain and to other NADH producing pathways. We showed that the [NAD^+^]/[NADH] ratio affects the accumulation of CoA esters via the close-to-equilibrium reaction M/SCHAD. It is plausible, therefore, that individual differences in the activity of the respiratory chain modulate the risk of substrate overload.

*A link to CoA metabolism–*The depletion of free CoA closed the vicious cycle. The relatively low capacity of MCKAT, accumulation of its ketoacyl-CoA substrates, and amplification of CoA-ester accumulation by the low equilibrium constant of M/SCHAD led to a sequestration of CoA in these esters. This limited MCKAT further, leading to even more sequestration of CoA. Yet, we never observed a complete depletion of CoA in the model. In fact, complete depletion would be incompatible with mFAO flux, since both MCKAT and CPT2 require CoA as a substrate. This crucial role of CoA suggests that CoA-releasing reactions may protect the mFAO pathway. Examples of CoA releasing processes are the acyl-CoA hydrolysis by thioesterases [26–28] or the ketogenesis pathway branching from C4-ketoacylCoA (acetoacetyl-CoA) via the enzyme HMG-CoA synthase towards ketone bodies. With respect to thioesterases, Eaton already described their potential mFAO-protecting role, but without a vicious cycle he had no reason to believe that the CoA concentration could decrease so dramatically [21]. Expression of mitochondrial acyl-CoA thioesterases in the liver is upregulated on a high-fat diet, fasting and regulated by the peroxisome-proliferator activated receptors (PPARs) [27,29], suggesting that they indeed play a role under fatty-acid overload conditions. Among the mitochondrial acyl-CoA thioesterases (ACOTs) the ACOT9 isoenzyme has the broadest chain-length specificity. It is the only ACOT that catalyses the hydrolysis of short- and medium-chain acyl-CoA esters [28], making it optimally suited to protect against the vicious cycle. Moreover, ACOT9 is strongly inhibited by CoA [28], ensuring that the ATP-dependent futile cycle of acyl-CoA synthesis and hydrolysis is only activated when CoA is low. Surprisingly, ACOT9 expression in liver is relatively low, at least in mice on a chow diet [28]. It will be interesting to see if it is induced on a high-fat diet, during fasting, or in mFAO disorders. With respect to ketogenesis, the production of ketone bodies from acetoacetyl-CoA or acetyl-CoA and their subsequent export from the liver also liberate CoA. Importantly, enzymes catalysing reactions of the ketogenic pathway, are both upregulated and post-transcriptionally activated during fasting [30] when the liver depends on mFAO. Finally, the first enzyme of the ketogenesis pathway starting from acetyl-CoA (acetoacetyl-CoA thiolase, encoded by ACAT1, which is highly expressed in the liver [31]), is reversible and may also work in the direction of beta-oxidation[32,33], thus providing a bypass for MCKAT.

In contrast, activation of CoA-utilizing pathways in the mitochondria may decrease the free CoA concentration and thereby trigger acute failure of mFAO. Mitchell [34] hypothesized that this may be a common denominator in all enzyme defects that lead to accumulation of CoA esters. He termed this group of diseases *C*o*A S*equestration, *T*oxicity and *R*edistribution (CASTOR) diseases and argued that they all share the risk of hypoglycemia. A surplus of a CoA-dependent substrate may then be sufficient to trigger the vicious cycle in patients with a mFAO disease.

It is tempting to speculate that the activity of the CoA synthesis pathway [35–37] may modulate the risk of CoA depletion. Indeed, increasing the total CoA pool (esterified plus free) does increase the threshold concentration of palmitoyl-CoA at which the flux decline sets in (Figure in S9 Fig). However, this comes at the cost of increased CoA-ester concentrations. As these may become toxic, we doubt that CoA biosynthesis may provide a strong protective mechanism.

Finally, the transesterification of CoA esters into carnitine esters and their subsequent excretion may protect cells against CoA depletion. Indeed, carnitine esters derived from most mFAO intermediates, including hydroxyacyl-carnitine esters, were found in serum and urine of mFAO patients [3,4]. It has been shown that this can lead to low circulating carnitine concentrations in mice and patients with mFAO defects [38–41]. Therefore, patients sometimes receive carnitine suppletion. Extra carnitine should stimulate the export of mFAO intermediates in the form of carnitine esters via the CACT transporter and thereby stimulate regeneration of CoA. The effectiveness of carnitine supplementation to patients is debated, though [42]. The carnitine concentration in blood does not reflect that in tissues [43] and there are also reports of acyl-carnitine-mediated toxicity [44]. Moreover, carnitine supplementation might also stimulate mitochondrial fatty-acid uptake via the CPT1-dependent shuttle, thereby aggravating the situation.

*Formation of enzyme supercomplexes–*With the exception of the reactions catalysed by MTP [45–51], our computational model did not include the formation of enzyme complexes. In such complexes intermediate metabolites may be channelled from one active site to another. In reality, however, also other enzymes may form complexes and these may affect the pathway kinetics. For instance M/SCHAD has been found in a respiratory supercomplex with the NADH dehydrogenase Complex I [52,53]. In the light of our modelling it seems plausible that this facilitates a direct channelling of NADH to the respiratory chain. This would push the M/SCHAD equilibrium in the forward direction and relieve the accumulation of CoA esters. Furthermore, we cannot exclude that acyl-CoA esters are partly channelled from MTP to the acyl-CoA dehydrogenases, since these enzymes have all been found in supercomplexes [54]. This would reduce the soluble acyl-CoA concentrations in the mitochondrial matrix and thereby relieve the product inhibition of MCKAT. There exists to our knowledge, however, no direct evidence for such channeling and the observation of high acyl-carnitine concentrations in patients’ serum is rather interpreted as a reflection of high acyl-CoA concentrations in the tissues. Finally, M/SCHAD has been found in complex with mitochondrial HMG-CoA synthase, the second enzyme of ketogenesis [55]. This association might channel C4-ketoacyl-CoA directly into ketone bodies. Such a mechanism would safeguard ketone-body synthesis and at the same time reduce the pressure on MCKAT.

*Concluding remarks–*It is clear that the mFAO pathway is connected to other metabolic pathways at crucial branch points. To get a full overview of the potential biomedical consequences of the connections and to make the model suitable for clinical application, it needs to be extended and further validated under a range of clinically relevant conditions. Nevertheless, even the model of the isolated mFAO pathway gives us unprecedented insight into potential risk factors and protection mechanisms. Future research should elucidate to what extent these may explain the patient-to-patient variation and the acute triggers leading to metabolic failure.

## Methods

### Computational models

All analyses were done with the computational model of mFAO as published in [8]. Calculations were done in Wolfram Mathematica (Wolfram Research, Inc., Champaign, IL, USA), version 10.2.0.0. A full description of the model equations is given in [8]. The model itself can be found at https://jjj.bio.vu.nl/models/vaneunen6. Initial conditions for steady-state calculations were the steady-state metabolite concentrations at a cytosolic palmitoyl-CoA concentration ([palmitoyl-CoA]_CYT_) of 0.1 μM. The Mathematica functions NDSolve and FindRoot were used for the time simulations and to find steady states, respectively, as done in [8]. As mFAO flux we took the uptake flux of palmitoyl-carnitine into the mitochondria (J_uptake_), which is the steady-state rate of the C16-palmitoyl-carnitine uptake through CACT. The scripts for removing promiscuity and for computing the regulatory contributions ${\left|\bar{\Theta }\right|}_{X_{j}}^{v}$ are given in S1 Appendix and S2 Appendix.

### Removal of promiscuity

Promiscuity was removed either from all enzymes in the model simultaneously or per enzyme, as indicated in the text. A similar procedure was followed in [8] for all enzymes simultaneously. To remove promiscuity from an enzyme, we first removed the substrates and products of other chain lengths, which are in the equation as competitive inhibitors (the grey-highlighted metabolites in Eq 9). Secondly, the enzyme concentration was partitioned evenly over the chain-length-specific reactions that it catalyses by adding a partition factor α to the rate equation. The value of α was exactly the same for all the reactions catalyzed by an enzyme; its value is 1 divided by the number of reactions catalyzed by the enzyme. In our previous study [8] we had based the partition factors α on the flux distribution in the original model, at a reference concentration of 25 μM of palmitoyl-CoA. The two ways of partioning yielded very similar results. An example will be given for the C16-specific acyl-CoA dehydrogenation reaction catalysed by VLCAD. The rate equation for this reaction in the original model with promiscuity is:

$$v_{VLCAD,C16}=\frac{sf_{VLCAD,C16}\cdot V_{max,VLCAD}\cdot \left(\frac{\left[C16-acyl-CoA\right]}{Km_{VLCAD,C16-acyl-CoA}}\cdot \frac{\left[FAD\right]}{Km_{VLCAD,FAD}}-\frac{\left[C16-enoyl-CoA\right]}{Km_{VLCAD,C16-acyl-CoA}}\cdot \frac{\left[FADH_{2}\right]}{Km_{VLCAD,FAD}\cdot Keq_{VLCAD}}\right)}{\left(1+\frac{\left[C16-acyl-CoA\right]}{Km_{VLCAD,C16-acyl-CoA}}+\frac{\left[C16-enoyl-CoA\right]}{Km_{VLCAD,C16-enoyl-CoA}}+\sum_{n=12}^{n=14}\frac{\left[Cn-acyl-CoA\right]}{Km_{VLCAD,Cn-acyl-CoA}}+\frac{\left[Cn-enoyl-CoA\right]}{Km_{VLCAD,Cn-enoyl-CoA}}\right)\cdot \left(1+\frac{\left[FAD\right]}{Km_{VLCAD,FAD}}+\frac{\left[FADH_{2}\right]}{Km_{VLCAD,FADH_{2}}}\right)}$$ (9)

The rate equation for the same reaction in the model without promiscuity became:

$$v_{VLCAD,C16}=\frac{\alpha \cdot sf_{VLCAD,C16}\cdot V_{max,VLCAD}\cdot \left(\frac{\left[C16-acyl-CoA\right]}{Km_{VLCAD,C16-acyl-CoA}}\cdot \frac{\left[FAD\right]}{Km_{VLCAD,FAD}}-\frac{\left[C16-enoyl-CoA\right]}{Km_{VLCAD,C16-acyl-CoA}}\cdot \frac{\left[FADH_{2}\right]}{Keq_{VLCAD}}\right)}{\left(1+\frac{\left[C16-acyl-CoA\right]}{Km_{VLCAD,C16-acyl-CoA}}+\frac{\left[C16-enoyl-CoA\right]}{Km_{VLCAD,C16-enoyl-CoA}}\right)\cdot \left(1+\frac{\left[FAD\right]}{Km_{VLCAD,FAD}}+\frac{\left[FADH_{2}\right]}{Km_{VLCAD,FADH_{2}}}\right)}$$ (10)

### Metabolic control analysis

All enzyme rate equations in the model are of the shape:

$$v={sf}_{Cn}∙V_{max}∙f(X)$$ (11)

in which *v* is the rate of the enzyme, *sf*_*Cn*_ a specificity factor which corrects the maximal velocity *V*_*max*_ for the carbon chain length of the substrate, and *f(****X****)* is a function of the metabolite concentrations in vector ***X***.

Flux control coefficients were approximated as follows:

$$C_{enzyme}^{J}=\frac{dlnJ/dp}{\partial lnv/\partial p}=\frac{dJ}{dV_{max,enzyme}}\cdot \frac{V_{max,enzyme}}{J}\approx \frac{\Delta J}{\Delta V_{max,enzyme}}\cdot \frac{V_{max,enzyme}}{J}$$ (12)

Here *p* denotes a parameter that only affects the rate of the enzyme that is analysed. For this purpose we used the *V*_*max*_, since the rates of all reactions were proportional to this parameter (Eq 11). For the finite change denoted by Δ both +0.0001% and -0.0001% were taken, and the result was accepted if the two gave identical results within 1%. The chain-length-specific factors were calculated as:

$$C_{enzyme,Cn}^{J}\approx \frac{\Delta J}{{\Delta sf}_{Cn}}∙\frac{{sf}_{Cn}}{J}$$ (13)

in which Δ*sf*_*Cn*_ was again changed by +0.0001% and -0.0001%. It was checked that the sum of the chain-length-specific flux control coefficients was equal to the overall flux control coefficient of the enzyme, and that the sum of all flux control coefficients in the model was equal to 1 [11,14].

Flux response coefficients were approximated as:

$$R_{p}^{J}=\frac{dlnJ}{dlnp}\approx \frac{\Delta J}{\Delta p}∙\frac{p}{J}$$ (14)

in which *p* again denotes a model parameter. In this case the finite change of the parameter value was taken to be +0.0001% and -0.0001%. The validity of the calculated control coefficients were checked with the summation theorem [11,14] and the response coefficient of the [NAD^+^]/[NADH] ratio was checked with the connectivity theorem [11,14]. The response coefficients were calculated for each parameter in the model, but only the highest values were shown.

### Quantification of regulation of enzyme rates by individual variable metabolites

The contribution of each metabolite to the regulation of a specific enzyme rate was stepwise calculated according to [12] as follows. First, we calculated:

$${\frac{dlnv_{i}}{dt}|}_{X_{j}}(t)=\varepsilon_{X_{j}}^{v_{i}}(t)∙\frac{d{lnX}_{j}}{dt}=\left(\frac{{\delta v}_{i}}{\delta X_{j}}(t)∙\frac{X_{j}(t)}{v(t)}\right)∙\frac{dX_{j}(t)}{dt}∙\frac{1}{X_{j}(t)}$$ (15)

where v_i_ denotes the rate at which a specific enzyme catalyses reaction i. The outcome was plotted in Fig 4B and Figure in S7 Fig.

For Fig 5A–5F, the corresponding absolute of these values (calculated with the Mathematica function “Abs”) were integrated over time (from t = 0 through 400 minutes, which was enough to reach a new steady state under all inspected conditions) with the Mathematica function “NIntegrate” and divided by 400 minutes. This gives the average absolute (total) contribution of each metabolite (${\left|\bar{\Theta }\right|}_{X_{j}}^{v}$). In the net contribution of a metabolite the negative and positive contributions partially cancel each other out, since the positive areas under curve and the negative areas under curve are summed. However, in the average absolute contribution (as also calculated by the authors of this method), a real impression is given on how much a metabolite has actually contributed to the transition from the state at t = 0 to the new steady state since both positive and negative areas are made positive and then summed. This total contribution has been used for Fig 5A–5F. In Fig 5A–5F they are depicted as percentages of the sum of all total contributions of the metabolites that contributed to the rate in question, according to:

$$\frac{{\left|\bar{\Theta }\right|}_{X_{j}}^{v}}{\sum_{j}{\left|\bar{\Theta }\right|}_{X_{j}}^{v}}$$ (16)

Therefore, the relative average absolute contributions of all the contributing metabolites per reaction sum up to one:

$$\sum_{j}\frac{{\left|\bar{\Theta }\right|}_{X_{j}}^{v}}{\sum_{j}{\left|\bar{\Theta }\right|}_{X_{j}}^{v}}=1$$ (17)

## Supporting information

S1 Fig Model versions with full or limited promiscuity. **(A)** Steady-state uptake flux (J_uptake_, i.e. J_cactC16_) versus [palmitoyl-CoA]_CYT_ in the published model, the model without promiscuity and the 9 other models with each one enzyme without promiscuity. **(B)** J_uptake_ versus [palmitoyl-CoA]_CYT_ in the published model and the model without promiscuity at MCKAT for a concentration range from 0 till 500 μM.
(TIF)

S2 FigFlux control coefficients.**(A)** Flux control coefficients of all enzymes in the model on J_uptake_. **(B)** Flux control of chain-length specific MCKAT-catalysed reactions on J_uptake_. The sum of these chain-length specific control coefficients equals the overall $C_{MKAT}^{J_{uptake}}$, which is plotted in panel A.(TIF)

S3 Fig Steady-state CoA ester concentrations. Steady-state concentrations of mitochondrial acyl-CoA **(A)**, enoyl-CoA **(B)**, hydroxyacyl-CoA **(C)** and ketoacyl-CoA esters **(D)** as a function of [palmitoyl-CoA]_CYT_, specified per chain length.
(TIF)

S4 Fig Time course of mitochondrial CoA ester concentrations. Time course of mitochondrial concentrations of acyl-CoA, enoyl-CoA, hydroxyacyl-CoA and ketoacyl-CoA after an upshift of [palmitoyl-CoA]_CYT_ from 0.1 to 25 μM **(A)** or 60 μM **(B)**.
(TIF)

S5 Fig Time course of the rates of C_n_-acyl-CoA production, dehydrogenation and C_n-2_-acyl-CoA production per chain length (thus per oxidation cycle) after an upshift of [palmitoyl-CoA]_CYT_ 0.1 to 25 μM. (TIF)

S6 Fig vMTPC8+vMCKATC8, vMCADC6+vSCADC6 and vMCKATC6 after an upshift of [palmitoyl-CoA]_CYT_ from 0.1 to 60 μM. (TIF)

S7 Fig Metabolite contributions to vMCKATC6 after a sudden [palmitoyl-CoA]_CYT_ increase from 0.1 to 60 μM. (TIF)

S8 Fig**Fraction of the active site occupied by each of the indicated metabolites, calculated for MCKAT (A), MCAD (B), and SCAD (C) as a function of [palmitoyl-CoA]_CYT_.** The occupancy (Occ) of an enzyme (E) by a metabolite X_j_ was calculated by:
$${Occ}_{E,X_{j}}=\frac{\frac{\left[X_{j}\right]}{{Km}_{{E,X}_{j}}}}{\left(1+\frac{\left[X_{j}\right]}{{Km}_{{E,X}_{j}}}+\sum_{k}\frac{\left[X_{k}\right]}{{Km}_{{E,X}_{k}}}\right)}$$(18)
where X_k_ denotes a metabolite that the enzyme can also bind in the same pocket as X_j_.(TIF)

S9 Fig The effect of varying total mitochondrial CoA concentration (CoA_t,m_) on flux decline. (TIF)

S1 Appendix Model without promiscuity. (NB)

S2 Appendix Calculation of average absolute regulatory contribution. (NB)
