# Supplementary figures and images for: The promiscuous enzyme medium-chain 3-keto-acyl-CoA thiolase triggers a vicious cycle in fatty-acid beta-oxidation
Source: PLoS Comput Biol. 2017 Apr 3;13(4):e1005461. doi: 10.1371/journal.pcbi.1005461 (PMC5397069; doi:10.1371/journal.pcbi.1005461)

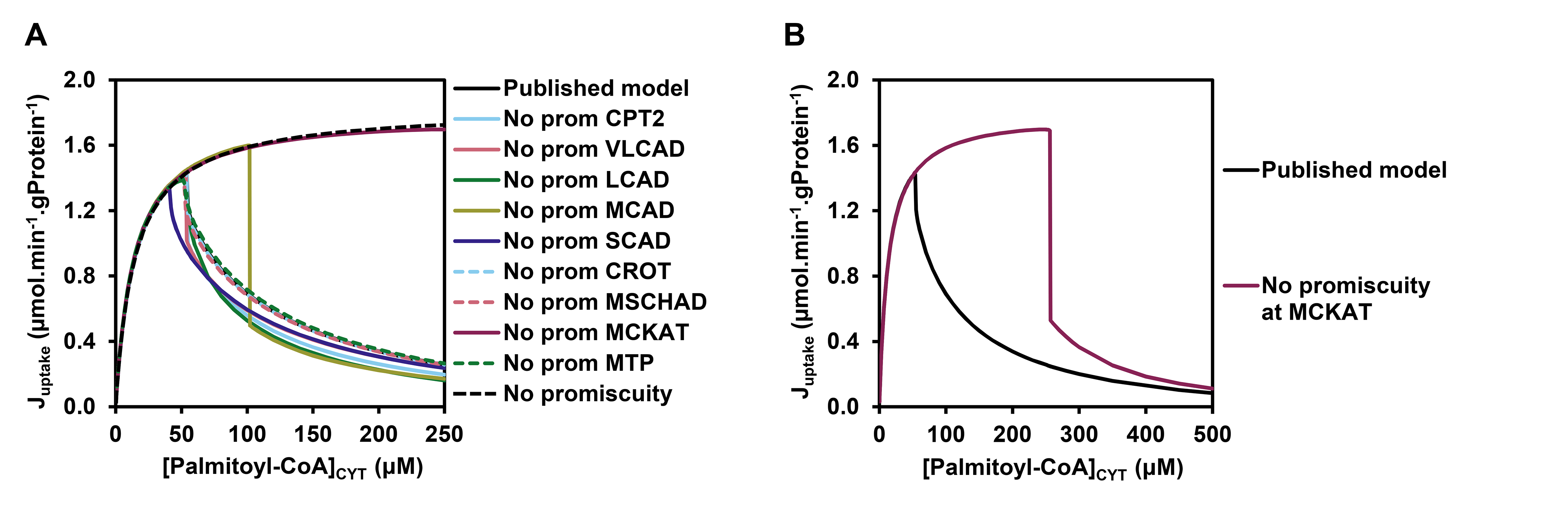

Supplement: S1 Fig — (A) Steady-state uptake flux (Juptake, i.e. JcactC16) versus [palmitoyl-CoA]CYT in the published model, the model without promiscuity and the 9 other models with each one enzyme without promiscuity. (B) Juptake versus [palmitoyl-CoA]CYT in the published model and the model without promiscuity at MCKAT for a concentration range from 0 till 500 μM. (TIF) [file pcbi.1005461.s001.tif]

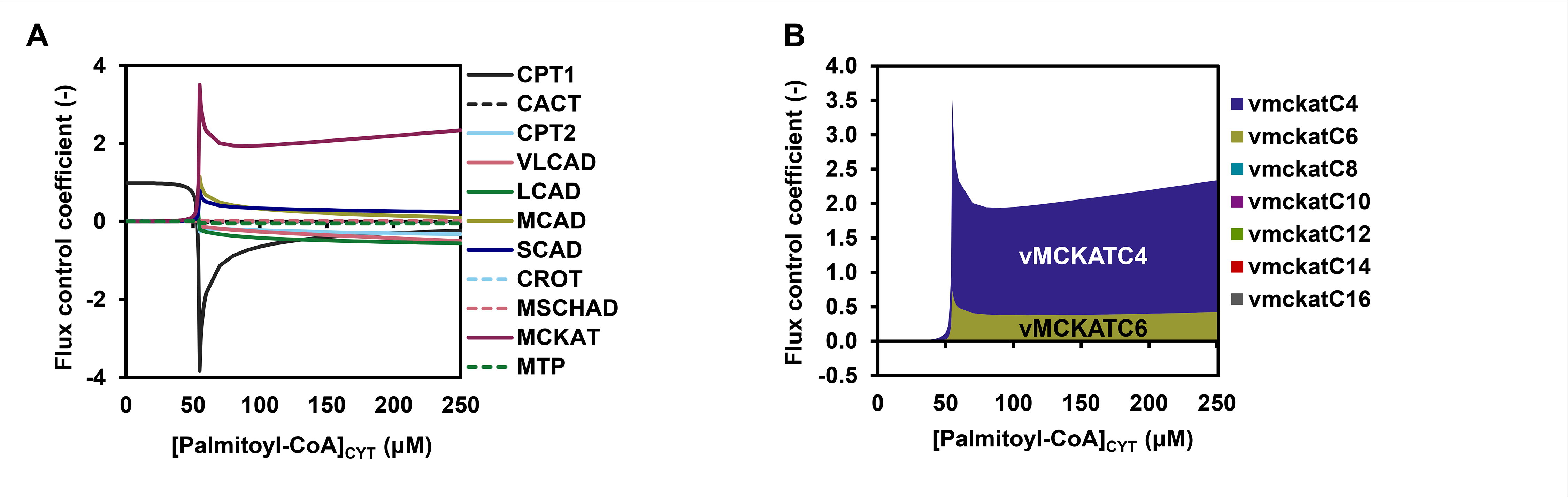

Supplement: S2 Fig — (A) Flux control coefficients of all enzymes in the model on Juptake. (B) Flux control of chain-length specific MCKAT-catalysed reactions on Juptake. The sum of these chain-length specific control coefficients equals the overall CMKATJuptake, which is plotted in panel A. (TIF) [file pcbi.1005461.s002.tif]

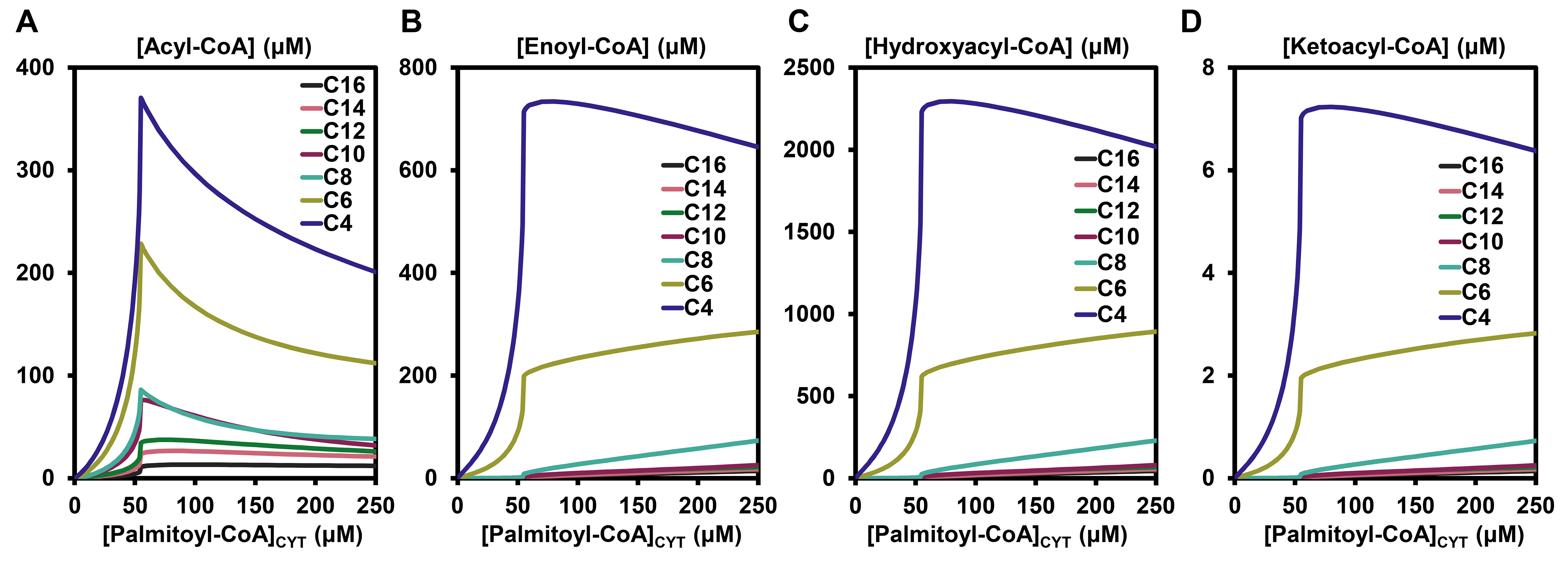

Supplement: S3 Fig — Steady-state concentrations of mitochondrial acyl-CoA (A), enoyl-CoA (B), hydroxyacyl-CoA (C) and ketoacyl-CoA esters (D) as a function of [palmitoyl-CoA]CYT, specified per chain length. (TIF) [file pcbi.1005461.s003.tif]

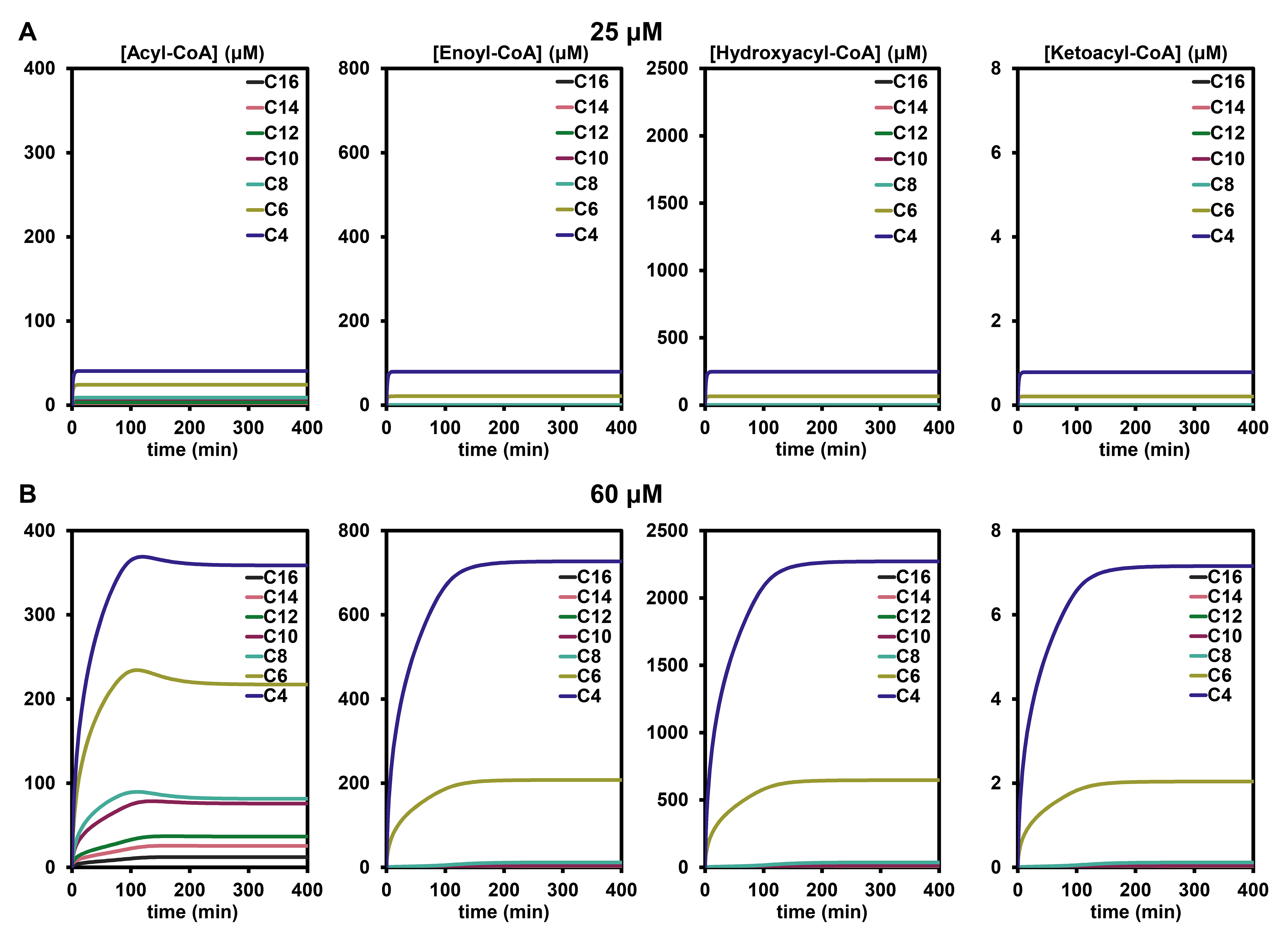

Supplement: S4 Fig — Time course of mitochondrial concentrations of acyl-CoA, enoyl-CoA, hydroxyacyl-CoA and ketoacyl-CoA after an upshift of [palmitoyl-CoA]CYT from 0.1 to 25 μM (A) or 60 μM (B). (TIF) [file pcbi.1005461.s004.tif]

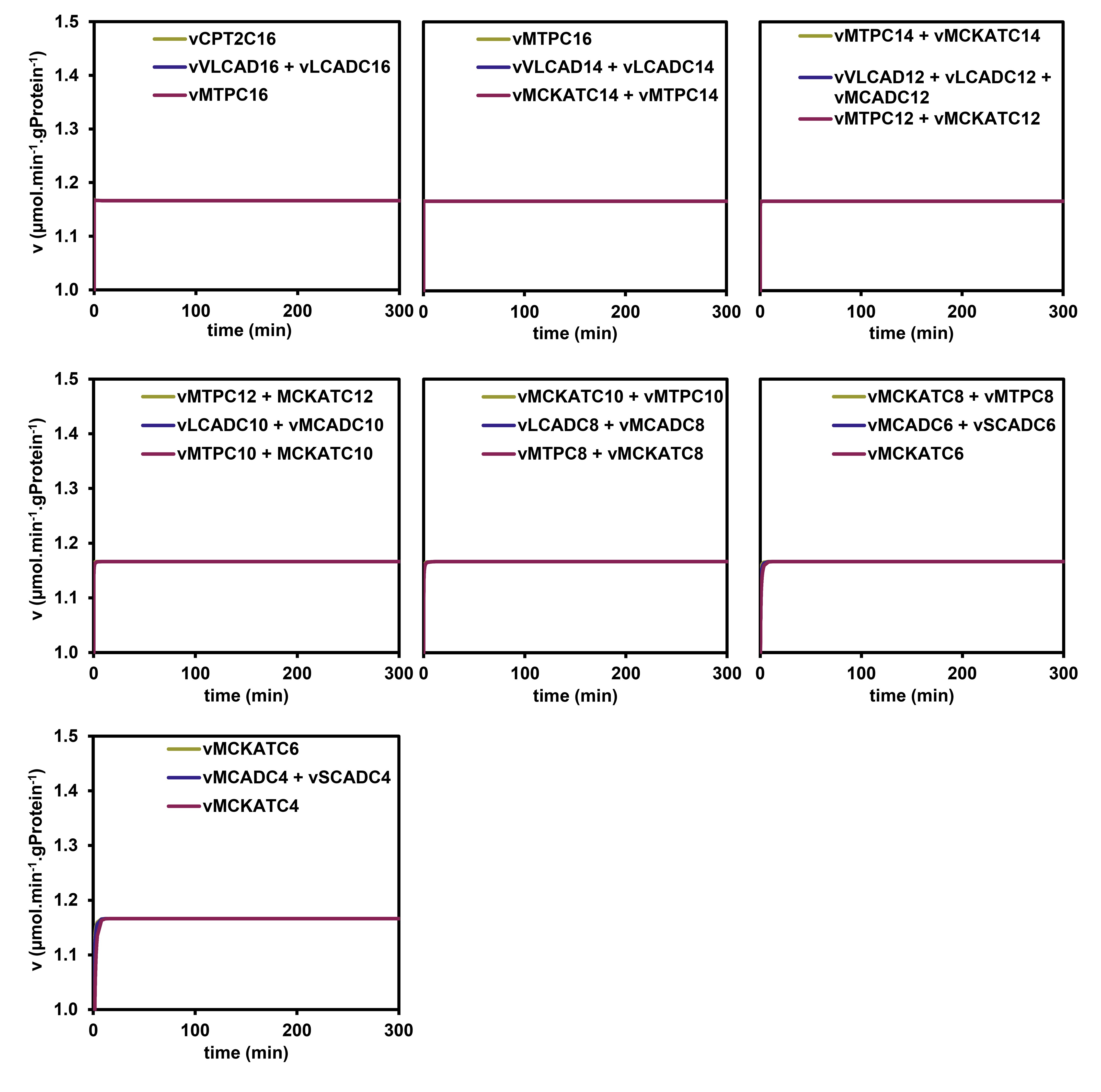

Supplement: S5 Fig — (TIF) [file pcbi.1005461.s005.tif]

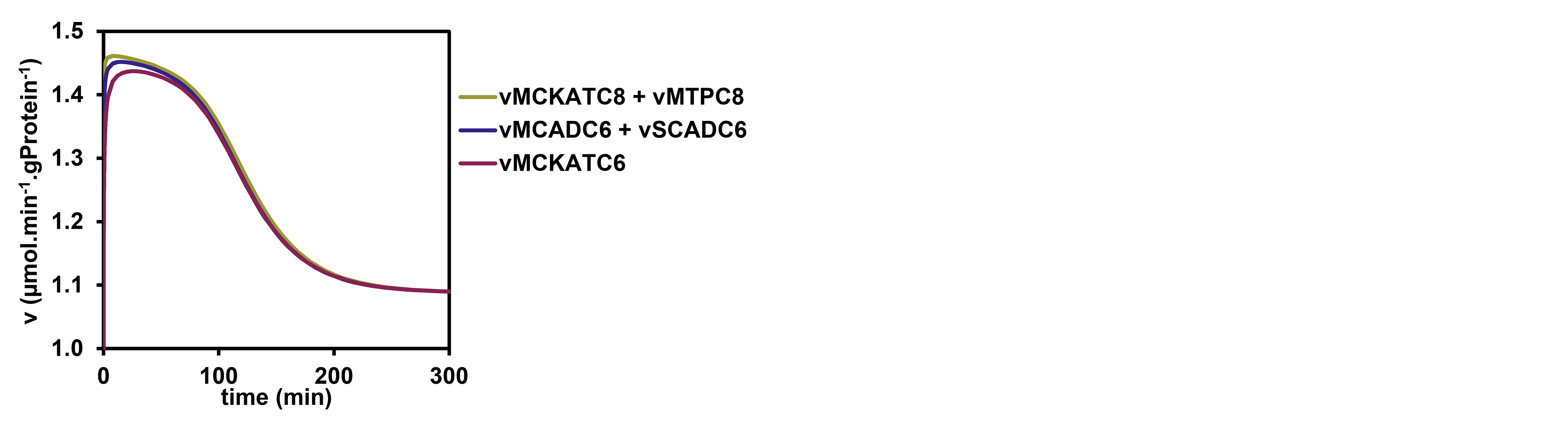

Supplement: S6 Fig — (TIF) [file pcbi.1005461.s006.tif]

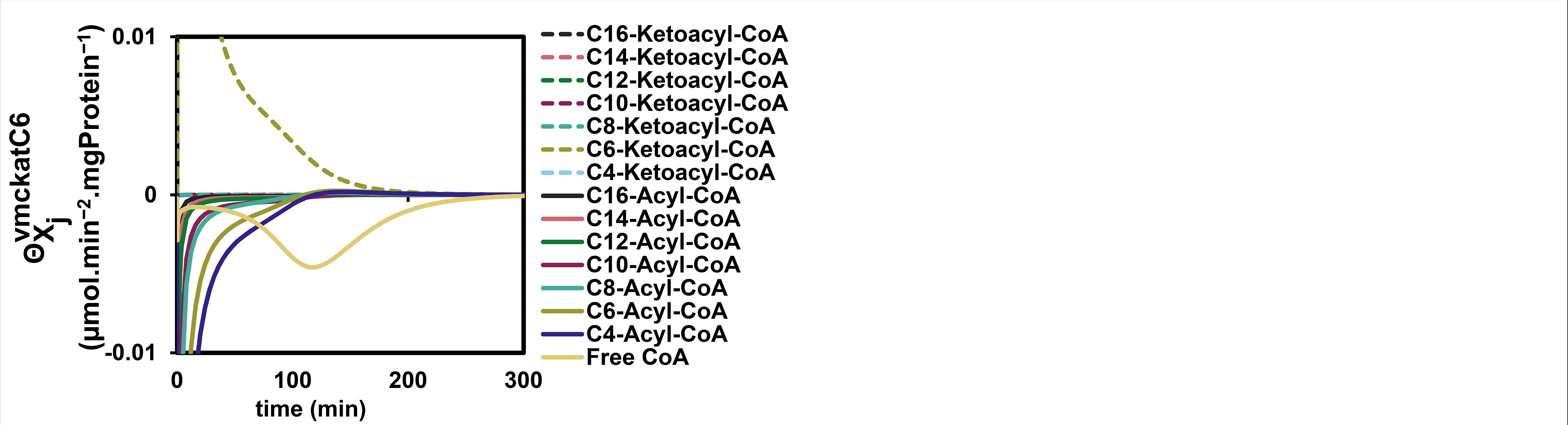

Supplement: S7 Fig — (TIF) [file pcbi.1005461.s007.tif]

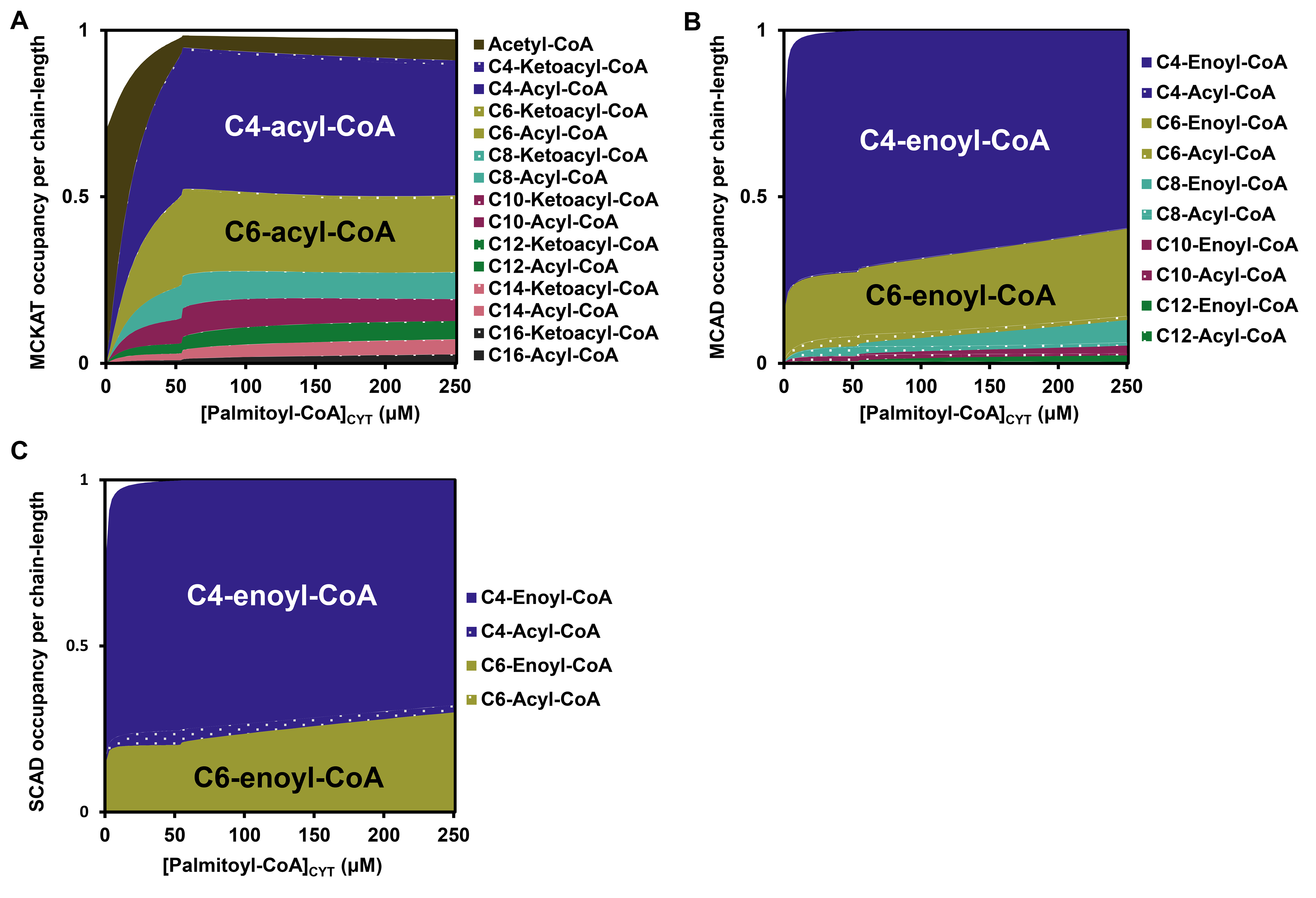

Supplement: S8 Fig — Fraction of the active site occupied by each of the indicated metabolites, calculated for MCKAT (A), MCAD (B), and SCAD (C) as a function of [palmitoyl-CoA]CYT. The occupancy (Occ) of an enzyme (E) by a metabolite Xj was calculated by: OccE,Xj=[Xj]KmE,Xj(1+[Xj]KmE,Xj+∑k[Xk]KmE,Xk)(18) where Xk denotes a metabolite that the enzyme can also bind in the same pocket as Xj. (TIF) [file pcbi.1005461.s008.tif]

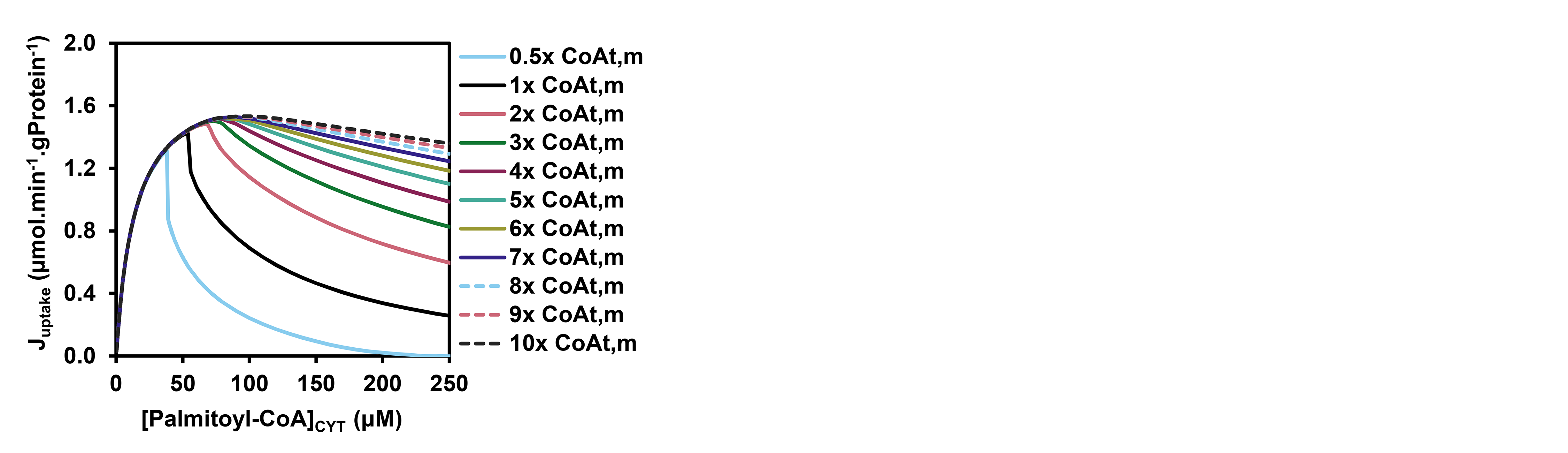

Supplement: S9 Fig — (TIF) [file pcbi.1005461.s009.tif]
